# Dynamic analysis of 22 serum biomarkers following pneumothorax

**DOI:** 10.3389/fmed.2026.1879882

**Published:** 2026-08-27

**Authors:** Jirui Chen, Qiang Lyu, Cheng Wang, Qianyu Han, Yuanyuan Wang, Mingwei Jiang, Lei Xue, Yuxiang Jin

**Affiliations:** Department of Thoracic Surgery, Second Affiliated Hospital of Naval Medical University, Shanghai, China

**Keywords:** dynamic changes, early diagnosis, pneumothorax, rat model, serum biomarkers

## Abstract

**Background:**

Pneumothorax is a common respiratory emergency. Current diagnosis primarily relies on imaging examinations, but these have insufficient sensitivity for early or occult cases, and there is a lack of highly specific molecular markers. Investigating the dynamic changes in serum biomarkers is of great significance for the early diagnosis and intervention of pneumothorax.

**Methods:**

A rat pneumothorax model was established. Serum was collected before modeling and at 1, 2, 6, 12, 24, and 48 h after modeling. Using pre-modeling rat serum marker levels as a baseline, 22 biomarkers (including inflammatory factors, fibrosis-related molecules, immunomodulatory molecules, and metabolic indicators) were detected using enzyme-linked immunosorbent assay (ELISA). Small animal ultrasound was used to verify model establishment.

**Results:**

The rat pneumothorax model was successfully established, with ultrasound showing the loss of the lung sliding sign. The biomarkers exhibited four dynamic patterns: (1) Early rapid increase in pro-inflammatory factors (tumor necrosis factor-*α*, interleukin-1*β*, etc.); (2) Sustained or fluctuating increase in fibrosis-related molecules (transforming growth factor-β, collagen, etc.); (3) Sustained decrease in the anti-inflammatory factor interleukin-10; (4) Fluctuating changes in metabolic indicators and aquaporin markers (25-hydroxyvitamin D, aquaporin-4/5). Notably, to our knowledge, this study is the first to discover early, significant, and rapid increases in tartrate-resistant acid phosphatase (TRACP), intercellular adhesion molecule-1 (ICAM-1), and nuclear factor-κB (NF-κB) in a pneumothorax model, which may provide a basis for the early diagnosis of pneumothorax.

**Conclusion:**

This study systematically delineates the dynamic evolution patterns of multiple classes of serum biomarkers following pneumothorax. The novel finding of abnormal elevations in TRACP, ICAM-1, and NF-κB challenges traditional understanding of their functions and provides new potential candidate targets for the early diagnosis of pneumothorax.

## 1 Introduction

Pneumothorax, as a common respiratory emergency, can lead to acute dyspnea, hypoxemia, and even circulatory failure, severely impacting patients’ health and quality of life. Clinically, many patients are diagnosed either incidentally through imaging studies while asymptomatic or when the condition progresses to life-threatening pneumothorax with respiratory and circulatory failure (1). Predicting recurrence during treatment remains challenging, with recurrence rates of approximately 30% for primary spontaneous pneumothorax (PSP) and as high as 45% for secondary spontaneous pneumothorax (SSP) (2). As clinical management strategies continue to evolve, individualized treatment for pneumothorax patients still requires more research evidence for support (3). Despite certain advancements in pneumothorax treatment, such as closed thoracic drainage and surgical intervention, early diagnosis remains a critical factor in reducing complication risks. Current clinical diagnosis primarily relies on imaging examinations (e.g., chest X-ray, computed tomography (CT)) and clinical symptom assessment. However, challenges persist in sensitivity and delayed judgment for early, mild, or complex cases. Therefore, identifying earlier and more precise molecular biomarkers is of significant importance for improving the early detection, risk assessment, and treatment strategy formulation of pneumothorax.

A genome-wide association study of inflammatory proteins in clinical patients suggested a potential causal relationship between inflammatory proteins and pneumothorax. Protective proteins identified include interleukin-17A (IL-17A) and sirtuin 2 (SIRT2), while risk-associated proteins include interleukin-13 (IL-13) and tumor necrosis factor-related apoptosis-inducing ligand (TRAIL). Multivariable Mendelian randomization analysis confirmed the robustness of these associations (4). Research on adolescent pneumothorax indicates that fine particulate matter accumulation, inflammatory responses (macrophage proliferation), and overexpression of monocyte chemoattractant protein-1 (MCP-1) and matrix metalloproteinase-9 (MMP-9) may be involved in the pathogenesis of primary spontaneous pneumothorax (PSP). Excessive accumulation of fine particulate matter may play a significant role in PSP occurrence among adolescents and young adults (5). However, the dynamic patterns of these molecules after pneumothorax and their clinical significance at different time stages have not been systematically elucidated. Existing studies are mostly limited to single or a few indicators, primarily focusing on comparative analysis between pneumothorax patients and healthy populations. The lack of overall analysis of the temporal characteristics of multiple indicators’ continuous changes hinders their translation into clinical applications.

Animal models are important tools for studying the pathophysiological mechanisms of pneumothorax. Among these, the rat pleural puncture-induced model is widely used due to its simple operation, good reproducibility, and ability to simulate the pathological changes of human pneumothorax (6, 7). With the advancement of clinical research, ultrasound has replaced chest X-ray as the first-line diagnostic tool for traumatic pneumothorax (sensitivity 91% vs. 47%), while CT is used for evaluating occult pneumothorax or planning surgical interventions (8). Consequently, this study employed ultrasound technology to verify the successful establishment of the animal model. Utilizing this model, we systematically detected the dynamic changes of 22 potential serum biomarkers at different time points (0, 1, 2, 6, 12, 24, 48 h) after pneumothorax induction. These biomarkers include inflammatory factors (IL-1*β*, IL-6, TNF-*α*), tissue repair-related molecules (TGF-β, MMPs, TIMPs), immunomodulatory molecules (PD-1). Among these, a particular focus was placed on the expression dynamics of TRACP, ICAM-1, and NF-κB for the following reasons: TRACP (tartrate-resistant acid phosphatase) was selected as a marker of macrophage and osteoclast activity, which is pivotal in the inflammatory remodeling of pleural and subpleural tissues following pneumothorax-induced injury. ICAM-1 (intercellular adhesion molecule-1) was prioritized due to its critical role in mediating leukocyte adhesion and transmigration across injured endothelium, a key early event following lung re-expansion. NF-κB (nuclear factor kappa-light-chain-enhancer of activated B cells) was chosen as a master transcriptional regulator, as its activation can be directly triggered by mechanical stretch and subsequently orchestrates the expression of multiple downstream inflammatory cytokines (e.g., IL-1β, IL-6). By analyzing these three markers, we aimed to capture inflammatory activity at the levels of cellular recruitment (TRACP), endothelial interaction (ICAM-1), and transcriptional control (NF-κB), thereby providing a more integrated assessment of the pathophysiology.

By screening biomarkers that show significant early elevation after pneumothorax and analyzing the dynamic characteristics of different biomarkers, this study aims to explore potential evidence that may inform early clinical diagnosis. It is expected to enhance the understanding of the pathophysiological processes of pneumothorax and may offer a more comprehensive molecular testing strategy for early warning and individualized treatment of pneumothorax. The target molecules identified in this study were selected from a pool of candidate molecules that, based on existing literature and preliminary experimental results, were considered potentially involved in inflammatory responses or tissue repair during pneumothorax progression.

## 2 Materials and methods

### 2.1 Establishment of the rat pneumothorax model

All animal husbandry and experimental procedures were conducted in accordance with the guidelines of the Animal Ethics Committee of Shanghai Changzheng Hospital. Male Sprague–Dawley (SD) rats (body weight 200-250 g; age 6–8 weeks) were provided by Shanghai Laboratory Animal Co., Ltd. Rats were housed in a standard specific pathogen-free (SPF) environment under constant conditions (temperature 22 ± 2 °C; humidity 50–60%; 12-h light/12-h dark cycle), with free access to food and water. Experiments commenced after one week of acclimatization.

Eight rats were used in this study. A pneumothorax model was established via transthoracic needle puncture and air injection, following this procedure: Rats were anesthetized with isoflurane, intubated, and connected to a small animal ventilator. Under ultrasound guidance, a needle was inserted at the lower edge of the right 5th intercostal space, and 1 mL of air was injected using a 5 mL syringe (6, 7). Studies indicate that key sonographic signs for pneumothorax diagnosis are the absence of lung sliding (the “barcode sign”) and the presence of a lung point (a specific sign for pneumothorax) (9). Therefore, successful model establishment was confirmed by observing the disappearance of respiratory lung motion under ultrasound. Subsequently, the puncture site was disinfected, anesthesia was stopped, and the rat was placed on a heating pad for recovery and close observation.

### 2.2 Blood sample collection after pneumothorax model establishment

Blood samples were collected from the rat retro-orbital plexus at baseline (before anesthesia) and at 1, 2, 6, 12, 24, and 48 h after pneumothorax modeling. The selected time points (1, 2, 6, 12, 24, and 48 h post-induction) were designed to capture the hyperacute (within 2 h), acute (6–12 h), and subacute (24–48 h) phases of the inflammatory and repair responses following pneumothorax, based on previous time-course studies of acute lung injury and pleural irritation models. Before collection, the eye area was locally anesthetized with 1% tetracaine, and approximately 0.2 mL of whole blood was collected using a heparinized capillary tube (diameter 1.0 mm). After collection, hemostasis was achieved using a sterile cotton ball, and chloramphenicol eye drops were administered to prevent infection. An equal volume of saline was supplemented after each sampling. Blood samples were incubated at room temperature for 30 min, then centrifuged at 3000 rpm for 15 min at 4 °C to separate serum, which was aliquoted into EP tubes and stored at −80 °C. After the final blood collection time point (48 h post-modeling), rats were humanely euthanized to avoid unnecessary suffering. Euthanasia was performed by inducing anesthesia with isoflurane followed by intraperitoneal injection of an overdose of pentobarbital sodium (150 mg/kg). Death was confirmed by the absence of heartbeat and pupillary reflex. Of the eight rats that underwent pneumothorax induction, two died before the 48-h endpoint due to surgical complications; thus, six rats completed the study and were included in the final analysis.

### 2.3 Enzyme-linked immunosorbent assay (ELISA) detection

Commercial ELISA kits were used to quantify the serum levels of 22 biomarkers according to the manufacturer’s instructions. Briefly, serum samples and standards were added to pre-coated 96-well plates, followed by incubation with detection antibodies and enzyme-conjugated secondary antibodies. Tetramethylbenzidine (TMB) substrate was added to initiate the color reaction, and absorbance was measured at 450 nm. All steps were performed in duplicate, with quality controls in place. A standard curve (R^2^ > 0.99) was established. Data were collected using a microplate reader (BioTek Synergy H1) and analyzed with GraphPad Prism 8.0 software. All ELISA kits were purchased from Jiangsu Enzyme Label Biotechnology Co., Ltd. (Jiangsu, China, Supplementary Table S1).

### 2.4 Ultrasound technology

Transthoracic echocardiography was performed on all surviving rats using a high-resolution small animal ultrasound system (VisualSonics Vevo 2,100) before and after pneumothorax modeling (i.e., after anesthesia and after puncture/air injection). Rats were anesthetized with 1.5% isoflurane, placed supine on a temperature-controlled platform, chest hair was removed, and ultrasound gel was applied. Standard lung ultrasound images were acquired using an MS250 probe (21 MHz).

### 2.5 Statistical analysis

Repeated measures analysis of variance (ANOVA) was employed, followed by LSD post-hoc tests to compare differences between time points. All data are presented as mean ± standard deviation. A *p*-value < 0.05 was considered statistically significant.

## 3 Results

### 3.1 Successful establishment of the pneumothorax model

**Figure 1:**
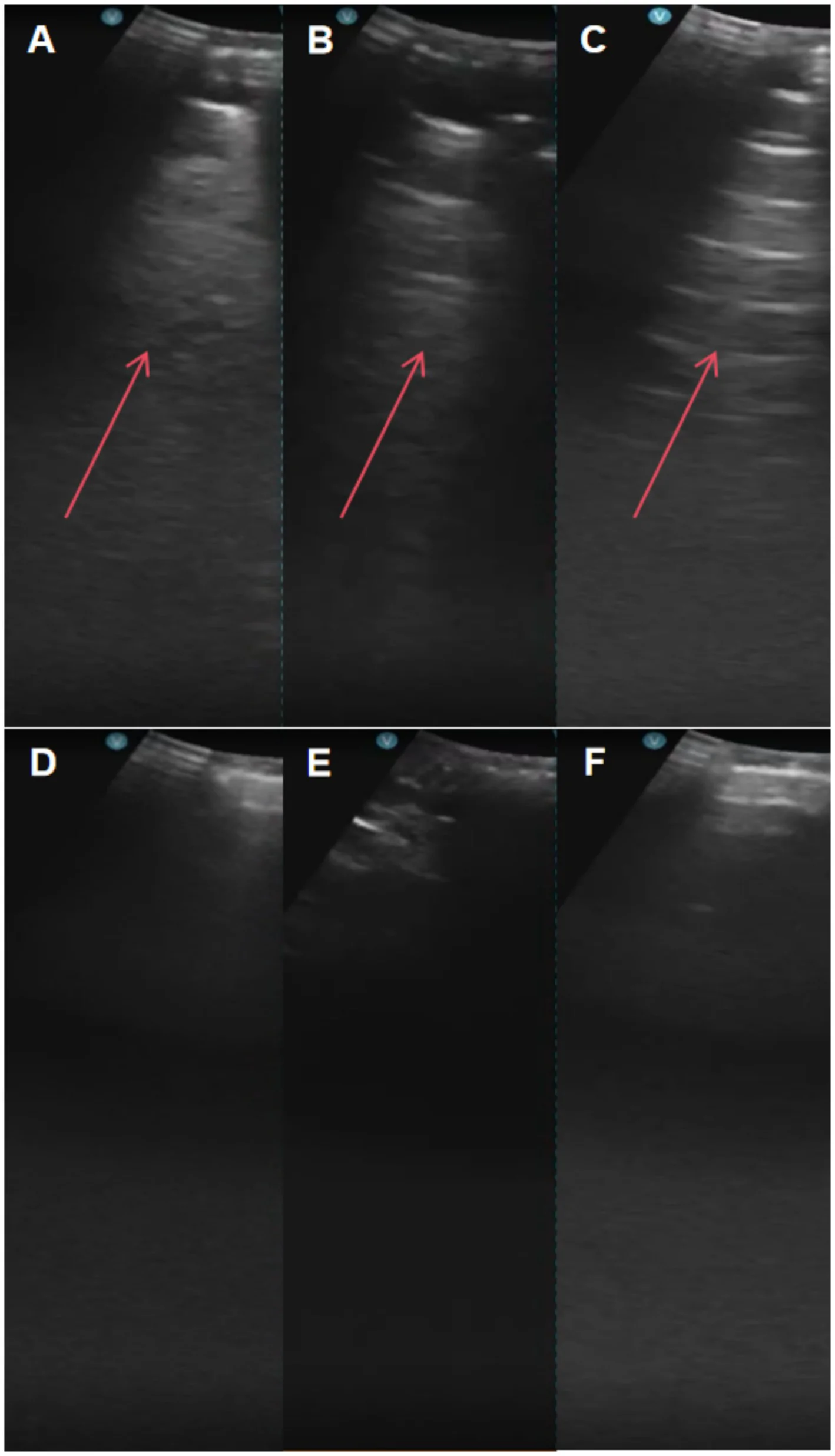
Comparison of ultrasound images before and after pneumothorax induction in rat model. **(A–C)** Show thoracic ultrasound images of three different rats before modeling. **(D–F)** Display thoracic ultrasound images of the same rats after modeling, corresponding to the images directly above them. The success of the model was confirmed by the disappearance of lung respiratory contraction observed under ultrasound after modeling.

The acute rat pneumothorax model was successfully established using the right thoracic wall puncture air injection method. Ultrasound images before and after modeling in rats showed a significant reduction in image changes caused by respiration in the pneumothorax area (Figures 1A–F), and the disappearance of the lung sliding sign (“barcode sign”) and lung point (a specific sign of pneumothorax) were observed. These images indicate that the pneumothorax model is stable and reliable, suitable for subsequent dynamic analysis of serum biomarkers.

### 3.2 Short-term rapid increase

**Figure 2:**
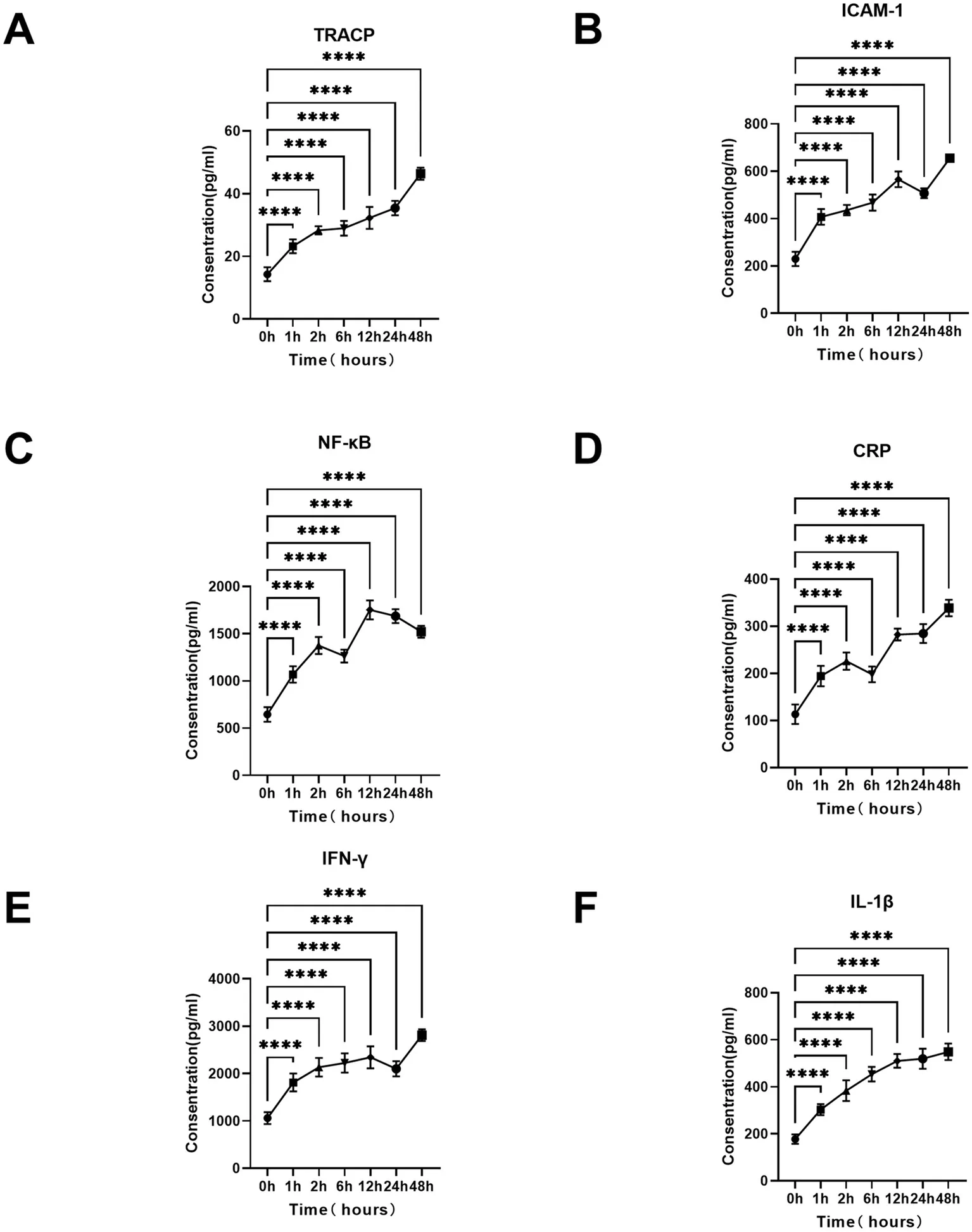
Protein markers showing rapid short-term increase (*n* = 6). **(A)** TRACP (baseline: 14.28 ± 2.22 pg./mL): increased to 23.23 ± 2.19 pg./mL at 1 h, reaching 46.42 ± 1.93 pg./mL at 48 h. **** represents *p* < 0.0001. **(B)** ICAM-1 (baseline: 229.59 ± 30.24 pg./mL): significantly increased to 407.18 ± 33.11 pg./mL at 1 h, reaching 655.03 ± 14.89 pg./mL at 48 h. **** represents *p* < 0.0001. **(C)** NF-κB (baseline: 645.43 ± 77.43 pg./mL): significantly increased to 1068.49 ± 86.79 pg./mL at 1 h, reaching 1522.33 ± 62.35 pg./mL at 48 h. **** represents *p* < 0.0001. **(D)** CRP (baseline: 113.12 ± 20.58 ng/mL): significantly increased to 194.21 ± 21.75 ng/mL at 1 h, continuously rising to 338.59 ± 17.51 ng/mL at 48 h. **** represents *p* < 0.0001. **(E)** IFN-*γ* (baseline: 1060.02 ± 125.40 pg./mL): significantly increased to 1812.96 ± 189.39 pg./mL at 1 h, reaching 2811.86 ± 123.50 pg./mL at 48 h. **** represents *p* < 0.0001. **(F)** IL-1*β* (baseline: 177.63 ± 20.07 pg./mL): rapidly increased to 302.68 ± 23.35 pg./mL at 1 h, reaching 549.16 ± 35.11 pg./mL at 48 h. **** represents *p* < 0.0001.

As shown in Figure 2, these markers significantly increased within 1 h and continued to rise throughout the observation period (*p* < 0.05). TRACP: Baseline level was 14.28 pg./mL, increased to 23.23 pg./mL 1 h after pneumothorax, a 62.7% increase from baseline, and further increased to 46.42 pg./mL at 48 h, a 225.0% increase from baseline, showing an overall continuous rapid upward trend, making it a key indicator of abnormal responses to acute pneumothorax injury. ICAM-1: Baseline level was 229.59 pg./mL, increased to 350.0 pg./mL 1 h after pneumothorax, and reached 655.03 pg./mL at 48 h, showing an overall continuous significant upward trend. NF-κB: Baseline level was 645.43 pg./mL, rapidly increased to 1068.49 pg./mL 1 h after pneumothorax, showing early rapid increase characteristics, and increased to 1522.33 pg./mL at 48 h, showing a continuous upward trend throughout. Other markers also showed significant increases at 1 h, reaching peaks at 48 h, showing a continuous upward trend: CRP baseline was 113.12 ng/mL, reached 338.59 ng/mL at 48 h, showing a linear continuous increase; IFN-*γ* baseline was 1060.02 pg./mL, reached 2811.86 pg./mL at 48 h, with a significant increase magnitude; IL-1β baseline was 177.63 pg./mL, reached 549.16 pg./mL at 48 h, with the most significant increase from 0–1 h.

### 3.3 Fluctuating increase

**Figure 3:**
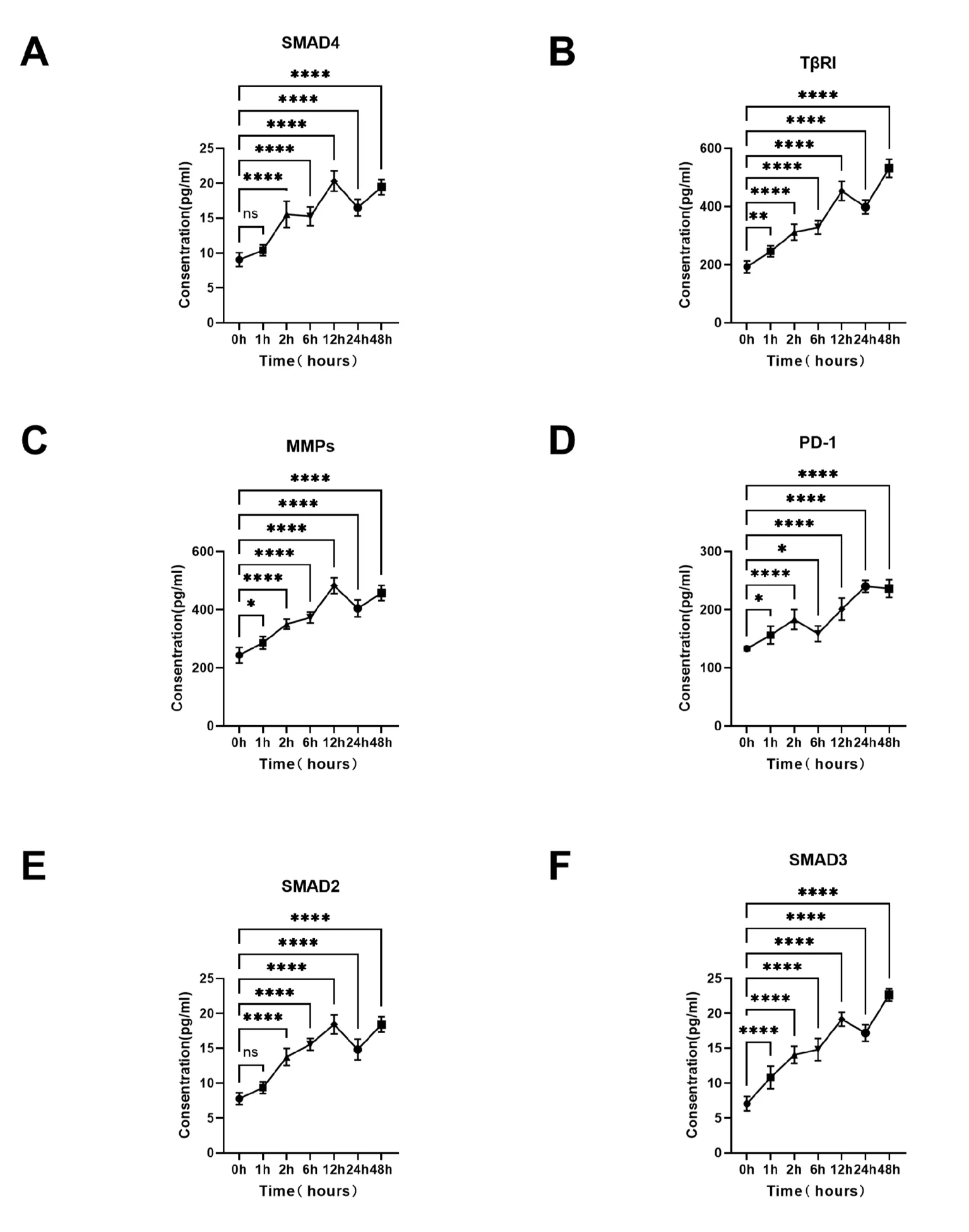
Protein markers exhibiting fluctuating elevation (*n* = 6). **(A)** SMAD4 (baseline: 9.05 ± 1.00 pg./mL): peaked at 12 h (20.30 ± 1.45 pg./mL), decreased to 16.50 ± 1.20 pg./mL at 24 h, then rose to 19.45 ± 1.10 pg./mL at 48 h. Ns represents not significant;**** represents *p* < 0.0001. **(B)** TβRI (baseline: 192.76 ± 20.41 pg./mL): Peaked at 12 h (453.43 ± 33.20 pg./mL), decreased to 398.43 ± 24.02 pg./mL at 24 h, then rose to 531.89 ± 31.08 pg./mL at 48 h. ** represents *p* < 0.01; **** represents *p* < 0.0001. **(C)** MMPs (baseline: 243.62 ± 26.87 ng/mL): peaked at 12 h (482.67 ± 26.89 ng/mL), decreased to 404.82 ± 28.98 ng/mL at 24 h, then rose to 457.44 ± 26.31 ng/mL at 48 h. *represents *p* < 0.05; ****represents *p* < 0.0001. **(D)** PD-1 (baseline: 133.03 ± 3.76 pg./mL): Peaked at 2 h (183.09 ± 16.94 pg./mL), decreased to 158.63 ± 13.53 pg./mL at 6 h, then rose to 236.33 ± 15.36 pg./mL at 48 h. *represents *p* < 0.05;**** represents *p* < 0.0001. **(E)** SMAD2 (baseline: 7.78 ± 2.14 pg./mL): no significant change at 1 h, mildly increased to 18.63 ± 2.45 pg./mL at 12 h, continuously rising to 18.91 ± 3.07 pg./mL at 48 h. Ns represents not significant; ****represents *p* < 0.0001. **(F)** SMAD3 (baseline: 7.06 ± 1.98 pg./mL): stable at 1 h, slowly increased to 17.72 ± 2.13 ng/mL at 12 h, significantly rose to 22.65 ± 1.89 pg./mL at 48 h. ****represents *p* < 0.0001.

Figure 3 illustrates that these markers showed fluctuating elevations, with levels at 48 h significantly higher than baseline (*p* < 0.05). Some markers showed a pattern of peaking at 12 h, decreasing at 24 h, and rebounding at 48 h: SMAD4, TβRI, MMPs, SMAD2, SMAD3 all exhibited this fluctuation characteristic, with an overall upward trend. SMAD4 baseline was 9.05 pg./mL, 48 h was 19.45 pg./mL; TβRI baseline was 192.76 pg./mL, 48 h was 531.89 pg./mL; MMPs baseline was 243.62 ng/mL, 48 h was 457.44 ng/mL; SMAD2 baseline was 7.78 pg./mL, 48 h was 18.91 pg./mL; SMAD3 baseline was 7.06 pg./mL, 48 h was 22.65 pg./mL. Some markers showed a pattern of peaking at 2 h, decreasing at 6 h, and significantly increasing at 48 h: PD-1 exhibited this fluctuation characteristic, with an overall upward trend; baseline was 133.03 pg./mL, 48 h was 236.33 pg./mL.

### 3.4 Short-term stability, increase

**Figure 4:**
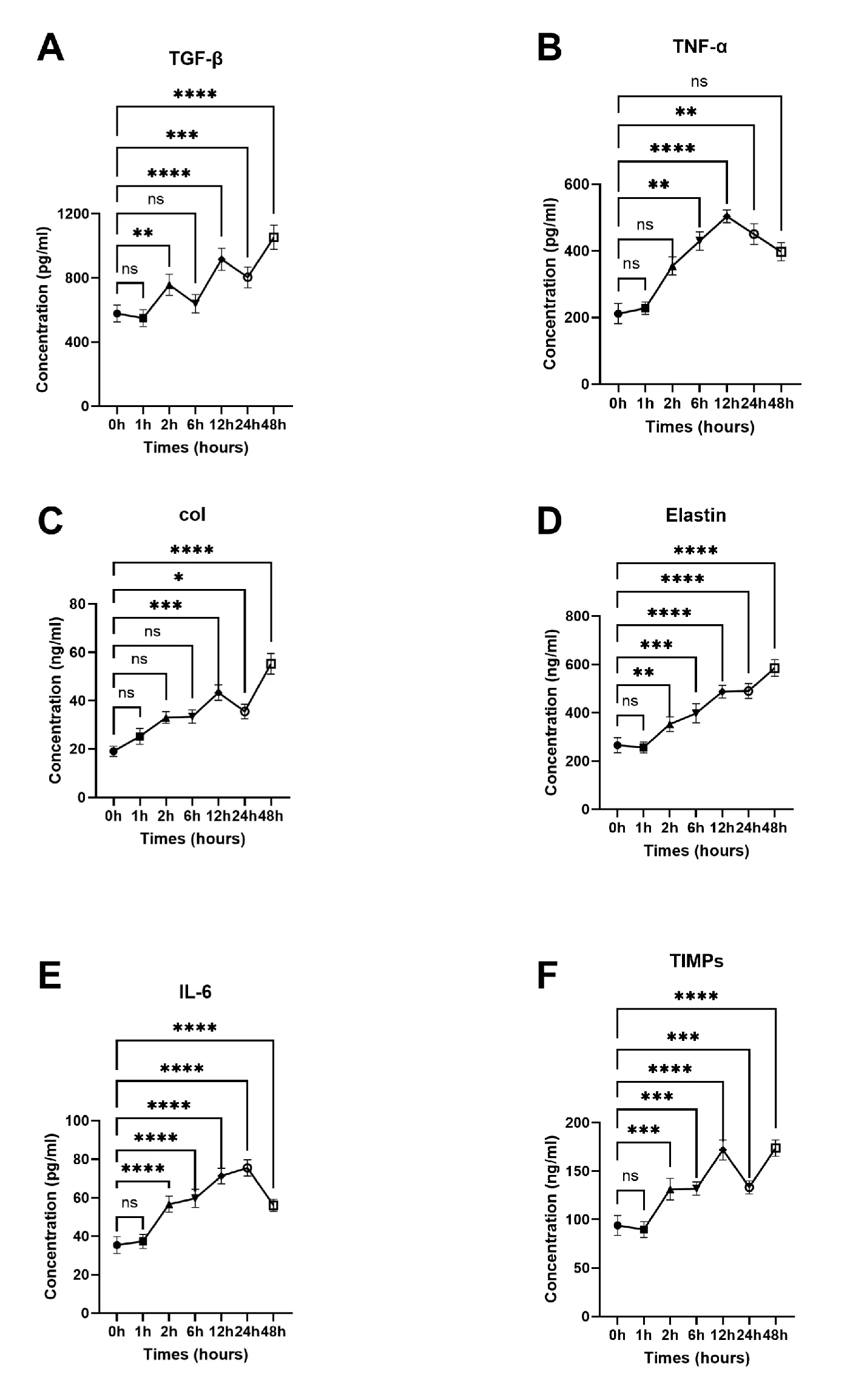
Protein markers demonstrating short-term stability with significant long-term changes (*n* = 6). **(A)** TGF-β (baseline: 578.10 ± 52.67 pg./mL): no significant change at 0–1 h, significantly increased to 757.41 ± 65.63 pg./mL at 2 h, reaching 1053.85 ± 75.31 pg./mL at 48 h. Ns re-presents not significant; ** represents *p* < 0.01; *** represents *p* < 0.001; **** represents *p* < 0.0001. **(B)** TNF-α (baseline: 211.98 ± 30.37 pg./mL): peaked at 12 h (504.41 ± 18.93 pg./mL), decreased to 450.87 ± 31.16 pg./mL at 24 h, further declined to 397.96 ± 27.26 pg./mL at 48 h. Ns represents not significant; ** represents *p* < 0.01; **** represents *p* < 0.0001. **(C)** Col (baseline: 19.07 ± 2.09 ng/mL): no significant change at 0–1 h, increased to 33.06 ± 2.45 ng/mL at 2 h, reaching 55.28 ± 4.26 ng/mL at 48 h. Ns represents not significant; * represents *p* < 0.05; *** represents *p* < 0.001; **** represents *p* < 0.0001. **(D)** Elastin (baseline: 265.56 ± 31.47 ng/mL): no significant change at 0–1 h, increased to 353.03 ± 30.78 ng/mL at 2 h, reaching 585.96 ± 34.23 ng/mL at 48 h. Ns represents not significant; ** represents *p* < 0.01; *** represents *p* < 0.001; **** represents *p* < 0.0001. **(E)** IL-6 (baseline: 35.42 ± 4.36 pg./mL): peaked at 24 h (75.54 ± 4.22 pg/mL), decreased to 56.12 ± 3.06 pg./mL at 48 h. Ns represents not significant, **** represents *p* < 0.0001. **(F)** TIMPs (baseline: 94.04 ± 10.21 ng/mL): no significant change at 0–1 h, increased to 131.34 ± 11.04 ng/mL at 2 h, reaching 173.75 ± 8.55 ng/mL at 48 h. Ns represents not significant; *** represents *p* < 0.001; **** represents *p* < 0.0001.

As depicted in Figure 4, these markers exhibited no significant change from 0 to 1 h but increased thereafter, with 48_h levels significantly higher than baseline (*p* < 0.05). Some markers showed no change from 0 to 1 h, increased at 2 h, and peaked at 48 h, as observed for TGF_β, Collagen, Elastin, and TIMPs, which all exhibited a continuous and steady increase, with TGF_β rising from a baseline of 578.10 pg/mL to a peak of 1053.85 pg/mL at 48 h, Collagen from 19.07 ng/mL to 55.28 ng/mL, Elastin from 265.56 ng/mL to 585.96 ng/mL, and TIMPs from 94.04 ng/mL to 173.75 ng/mL at 48 h; other markers showed no change from 0 to 1 h, peaked at 24 h, and decreased at 48 h, as seen for IL_6, which increased from a baseline of 35.42 pg/mL to a peak of 75.54 pg/mL at 24 h and then fell to 56.12 pg/mL at 48 h, still above baseline; and still other markers showed no change from 0 to 1 h, peaked at 12 h, and decreased at 48 h, as exemplified by TNF_α, which remained above baseline at 48 h (baseline: 211.98 pg/mL; 48 h: 397.96 pg/mL).

### 3.5 Fluctuation or decrease

**Figure 5:**
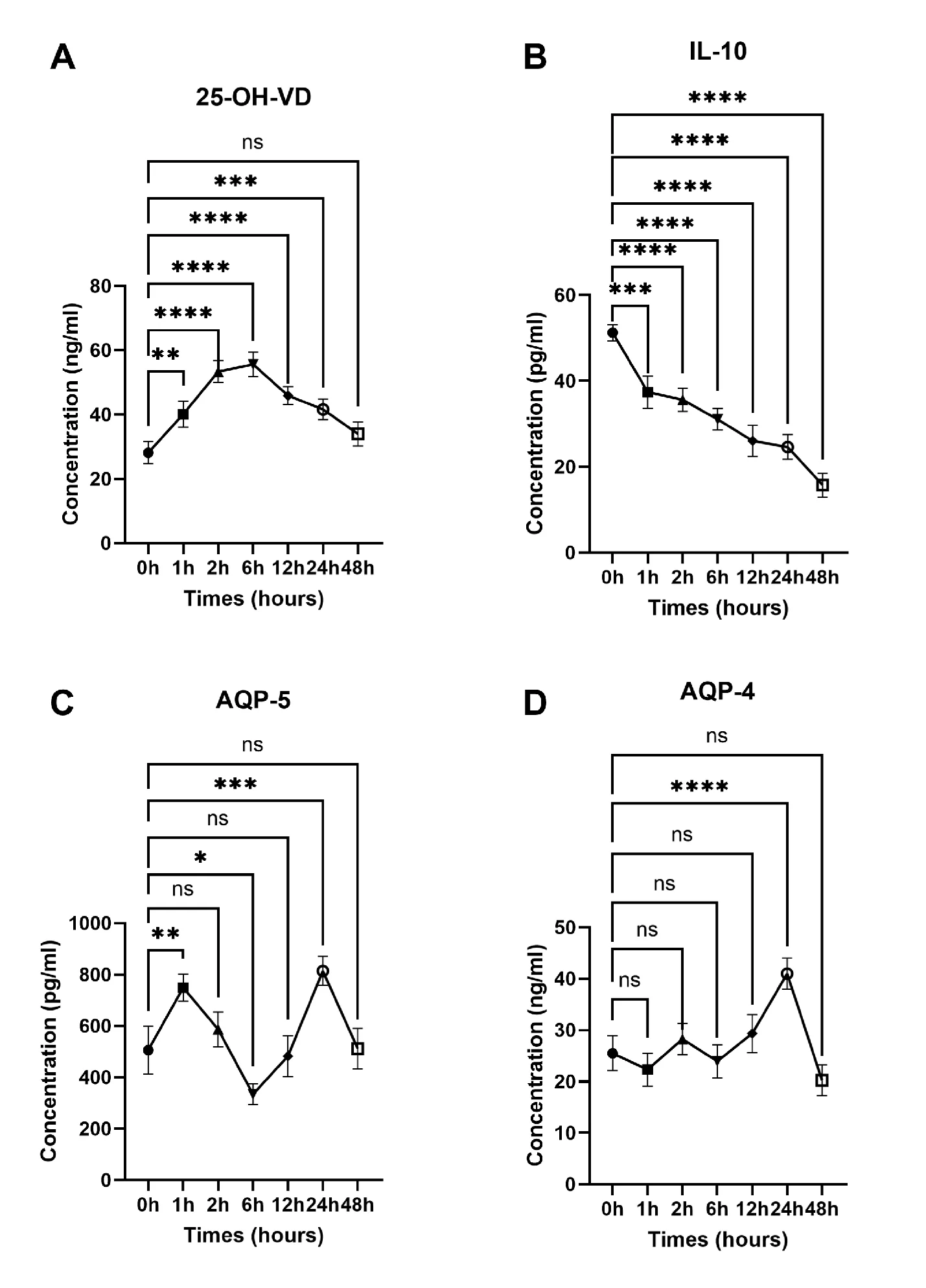
Protein markers with fluctuating changes or sustained decline (*n* = 6). **(A)** 25-OH-VD (baseline: 28.18 ± 3.45 ng/mL): peaked at 6 h (55.63 ± 3.79 ng/mL), decreased to 34.02 ± 3.74 ng/mL at 48 h. Ns represents not significant; ** represents *p* < 0.01; *** represents *p* < 0.001; **** represents *p* < 0.0001. **(B)** IL-10 (baseline: 51.26 ± 1.94 pg./mL): continuously decreased throughout the observation period, reaching 15.75 ± 2.77 pg./mL at 48 h. *** represents *p* < 0.001, **** represents *p* < 0.0001. **(C)** AQP-5 (baseline: 505.85 ± 93.25 pg./mL): increased to 749.57 ± 52.59 pg./mL at 1 h, decreased to 334.96 ± 40.82 pg./mL at 6 h, rose again to 814.74 ± 56.50 pg./mL at 24 h, then decreased to 512.32 ± 78.66 pg./mL at 48 h. Ns represents not significant; * represents *p* < 0.05; ** represents *p* < 0.01; *** represents *p* < 0.001. **(D)** AQP-4 (baseline: 25.85 ± 4.13 ng/mL): peaked at 24 h (41.03 ± 3.03 ng/mL), decreased to 20.27 ± 3.01 ng/mL at 48 h. Ns represents not significant, **** represents *p* < 0.0001.

Figure 5 shows that these markers displayed relative fluctuations, with significant changes (*p* < 0.05) at key time points. Peak followed by decrease: 25-OH-VD baseline was 28.18 ng/mL, peaked at 55.63 ng/mL at 6 h, decreased to 34.02 ng/mL at 48 h, close to baseline. Continuous decrease: IL-10 baseline was 51.26 pg./mL, showed a continuous decreasing trend throughout, decreased to 15.75 pg./mL at 48 h, each time point significantly lower than the previous time point. Frequent fluctuation throughout: AQP-5 baseline was 505.85 pg./mL, fluctuated repeatedly after pneumothorax, with frequent increases and decreases, 48-h level was 512.32 pg./mL, basically the same as baseline. Peak followed by decrease below baseline: AQP-4 baseline was 25.85 ng/mL, remained stable from 0–12 h, peaked at 41.03 ng/mL at 24 h, decreased to 20.27 ng/mL at 48 h, below baseline level.

## 4 Discussion

This study, by establishing a standardized rat pneumothorax model, for the first time systematically depicted the continuous dynamic profile of 22 serum biomarkers within 48 h post-injury. These biomarkers broadly cover key pathophysiological processes including inflammatory response, fibrosis and tissue repair, and immune regulation.

The inflammatory response is the primary pathophysiological change following pneumothorax (10). Lung collapse and pleural tear immediately release a large amount of damage-associated molecular patterns (DAMPs), activating the innate immune system and initiating the inflammatory cascade. TNF-*α* and IL-1β, as “initiators” and “core amplifiers” of inflammation, showed significant increases within 1 h after injury, primarily derived from activated alveolar macrophages and damaged epithelial cells (11, 12). TNF-α induces the expression of other inflammatory factors by activating signaling pathways such as NF-κB and mitogen-activated protein kinases (MAPKs), forming a positive feedback loop (13); IL-1β directly participates in the acute phase response, and its sustained high expression is related to the degree of tissue damage (14). CRP showed a significant increase within 1 h post-injury and continued to rise, indicating rapid and sustained activation of systemic inflammatory response, making it a reliable indicator for assessing the intensity of the acute phase response in pneumothorax (15). The increase in IFN-*γ* suggests early mobilization of the adaptive immune system (16).

In this study, pro-inflammatory factors (TNF-*α*, IL-1β, IL-6, CRP, IFN-γ) together with TRACP, ICAM-1, and NF-κB constituted a rapid-response, potent inflammatory network. Among them, the phenomenon of early significant rapid increase of TRACP, ICAM-1, and NF-κB in the pneumothorax model, to our knowledge, is the first report of this pattern and represents an important finding. All three showed significant increases within 1 h post-pneumothorax and continued to rise until 48 h, exhibiting highly synchronized kinetic characteristics. TRACP increased by 62.7% at 1 h compared to baseline and by 225.0% at 48 h; ICAM-1 increased by 52.4% at 1 h and by 185.3% at 48 h; NF-κB increased by 65.6% at 1 h and by 135.9% at 48 h. This synchronous activation suggests that the three may form a synergistic regulatory pathway in the early inflammatory response to pneumothorax, collectively mediating the pathophysiological response following acute injury.

TRACP is traditionally defined as a specific marker of osteoclast activity, widely used in the diagnosis and monitoring of bone metabolic diseases such as osteoporosis and bone tumors, and its role in acute thoracic injury has not been reported (17). This study is the first to confirm that TRACP exhibits early rapid response characteristics similar to classic inflammatory markers after pneumothorax, revealing its potential role in cross-system pathological responses. TRACP increased by 62.7% at 1 h and 225.0% at 48 h compared to baseline. The traditional function of ICAM-1 is mediating adhesion and migration of leukocytes and endothelial cells, being a key marker of endothelial cell activation (18). The innovative significance of its continuous increase in this study lies in first clarifying its dynamic change pattern in the early inflammatory response to pneumothorax. It not only shows early rapid activation, but the increase magnitude continues to expand, reflecting the sustained enhancement of leukocyte migration activity post-pneumothorax, suggesting endothelial cells play a core regulatory role rather than merely passive participation in pneumothorax inflammation. In existing research, NF-κB is a key transcription factor regulating cytokine gene expression, playing an important role in ischemia–reperfusion (IR)-induced acute lung injury (ALI). IR activates IKK-*β*, which phosphorylates IκB-*α*, leading to NF-κB release and nuclear translocation, thereby initiating pro-inflammatory gene transcription and amplifying the lung injury signaling chain (19). The innovative finding of this study lies in clarifying its early rapid activation characteristics (significant increase at 1 h) and sustained high expression until 48 h in acute pneumothorax injury, indicating that inflammatory signaling pathways are rapidly and continuously activated after pneumothorax, laying the molecular foundation for subsequent fibrosis processes, while also explaining the intensity and persistence of early inflammatory response in pneumothorax.

In sharp contrast to pro-inflammatory factors is the dynamic change of the anti-inflammatory factor IL-10. IL-10 showed a continuous monotonic decline from baseline to extremely low levels at 48 h, a discovery revealing insufficient anti-inflammatory compensatory response capacity. The imbalance between pro- and anti-inflammatory responses may lead to uncontrolled “inflammatory storm,” increasing the risk of systemic inflammatory response syndrome (SIRS) and even multiple organ dysfunction syndrome (MODS) (20). After lung collapse, the body immediately initiates repair programs to restore structural and functional integrity, with the core being the dynamic remodeling of the extracellular matrix (ECM) (21). TGF-*β* is the “core regulator” of tissue repair and fibrosis, showing a significant increase at 2 h post-injury, with an overall continuous upward trend (despite fluctuations). TGF-β comprehensively upregulates the synthesis of ECM components such as collagen (Col) and elastin by activating the SMAD signaling pathway (SMAD2/3/4, TβRI) and non-SMAD pathways (22, 23). The fluctuating increase patterns of SMAD2/3/4 and TβRI confirm the effective activation of this pathway post-pneumothorax, with fluctuations possibly reflecting negative feedback regulatory mechanisms. Collagen and elastin exhibited characteristics of “short-term stability, long-term continuous increase,” with no significant change initially, starting to rise steadily from 2 h post-injury, and peaking at 48 h, directly confirming the progress of the repair process (24, 25). Both MMPs and TIMPs showed synchronous fluctuating patterns of “increase - decrease - re-increase,” with this synchrony indicating the body’s fine regulation of ECM metabolic balance: early synchronous increase may clear damaged ECM while preventing excessive degradation; mid-phase decrease marks entry into a relatively stable repair stage; late re-increase may predict the initiation of pathological repair or fibrosis (26, 27). The local hypoxia, mechanical stress changes, and systemic inflammatory response caused by pneumothorax also affect the body’s metabolic homeostasis and fluid balance. 25-OH-VD showed a pattern of “first increase then decrease,” peaking at 6 h and gradually declining thereafter, with early increase possibly representing compensatory anti-inflammatory response, and later decrease suggesting compensatory exhaustion, providing an explanation for the association between vitamin D deficiency and poor pneumothorax prognosis (28). Frequent fluctuations of AQP-5 may be related to epithelial function during inflammation factor inhibition and repair processes. AQP-4 may reflect the central nervous system’s response to systemic stress or changes in blood–brain barrier function (7, 29).

We categorized the 22 biomarkers into three patterns. Early rapid continuous increase type (such as TRACP, ICAM-1, and NF-κB): sharply increase within 1–2 h and continue to rise, having potential for early diagnosis. Fluctuating increase type (such as SMADs, MMPs/TIMPs, IL-6, AQP-5): overall increase but with multi-stage fluctuations, reflecting complex feedback regulation and phase transitions, usable for disease staging and treatment response assessment. For example, peaks of IL-6 and 25-OH-VD may mark the inflammation peak; re-increase of SMADs may predict fibrosis initiation; persistently low AQP-5 may indicate pulmonary edema risk. Continuous decrease type (IL-10): progressive decrease indicates exhaustion of immune regulatory capacity and is a strong predictor of poor prognosis (30, 31). TRACP, ICAM-1, and NF-κB belong to the early rapid continuous increase type, suggesting potential clinical translational value. All three show significant increases within 1 h post-pneumothorax and may hold promise for improving early diagnostic sensitivity pending validation in clinical settings, and could be investigated as candidate bedside rapid diagnostic tools to complement imaging examinations. Among them, TRACP’s cross-system response characteristic makes it a unique supplementary indicator, combined with ICAM-1 (endothelial activation) and NF-κB (inflammatory switch), covering different levels of inflammatory response, potentially further improving diagnostic specificity. Their increase magnitude and rate are positively correlated with inflammation intensity, and their synergistic increase may serve as a quantitative indicator for assessing injury severity. The potential clinical relevance of dynamic systemic inflammation-based biomarkers in pneumothorax has been increasingly recognized. For instance, recent studies have demonstrated their adjunctive role in surgical risk stratification for first-episode primary spontaneous pneumothorax and in predicting mortality in post-pneumonectomy bronchopleural fistula, supporting the translational value of our observed biomarker dynamics (32, 33).

Although this study provides a comprehensive dynamic profile of serum biomarkers following pneumothorax, several limitations remain. The findings are derived from a rodent model, and their direct translational relevance to human pathophysiology requires further clinical validation. The relatively small sample size (*n* = 6) may limit the statistical power and generalizability of the results. The 48-h observation window captures only the acute phase and does not encompass long-term pathological processes such as chronic inflammation or fibrosis. Moreover, the observed associations between biomarker fluctuations and specific tissue events are correlative, and causal mechanistic links need to be confirmed through targeted intervention studies. In addition, the absence of a sham control group undergoing only anesthesia, mechanical ventilation, and thoracic puncture without air injection is a notable limitation. These procedural factors alone may induce certain inflammatory responses, and their potential confounding effects on the observed biomarker changes cannot be excluded. Future studies should incorporate sham-operated controls to isolate the specific effects of pneumothorax. The present study only covered a time window of 48 h after pneumothorax induction. Since all serum inflammatory markers continued to rise at 48 h and no descending phase was observed, the natural resolution time of the inflammatory response could not be determined. The lack of time points beyond 48 h (e.g., 72 h, 96 h, or longer) is a major limitation that prevented us from fully characterizing the dynamic evolution of inflammation. Future studies should extend the observation window to capture the peak and resolution phases of the inflammatory response.

Potential role of periostin in pneumothorax-induced lung injury. Periostin is a matricellular protein extensively studied in pulmonary diseases, with well-documented roles in airway inflammation, tissue remodeling, extracellular matrix regulation, and interstitial lung injury (34). Of particular relevance to the present findings, mechanical stretch and inflammatory cytokines (such as IL-6 and TGF-*β*, which were elevated in our experimental model) are known inducers of periostin expression in lung fibroblasts and epithelial cells (35, 36). Furthermore, periostin can activate NF-κB signaling and upregulate ICAM-1 expression, potentially creating a positive feedback loop that amplifies the inflammatory response to pneumothorax. Although periostin was not measured in the current study due to the predefined scope of our biomarker panel, these considerations suggest that future investigations should evaluate periostin levels—especially in patients with pneumothorax complicated by concomitant pulmonary infection or underlying interstitial lung disease—as it may offer more specific predictive value or reveal distinct pathophysiological subtypes. Given that this study is an exploratory animal experiment, the conclusions drawn are preliminary and require further confirmation in clinical samples and subsequent validation studies.

## 5 Conclusion

This study, through dynamic monitoring of 22 serum biomarkers, for the first time systematically mapped the time series of molecular events within 48 h post-pneumothorax. The abnormal increases in TRACP, ICAM-1, and NF-κB challenge traditional understanding of their functions and may, upon clinical validation, contribute to improving the sensitivity and specificity of early pneumothorax diagnosis. Integrating the dynamic data of TRACP, ICAM-1, and NF-κB with clinical and imaging parameters holds promise for establishing a more precise pneumothorax diagnostic mechanism. This study shifts the perspective of pneumothorax research from traditional imaging to molecular phenotype precision diagnosis and treatment. Future in-depth exploration of molecular synergistic regulatory mechanisms may make new contributions to the early diagnosis of pneumothorax.

## Data Availability

The raw data supporting the conclusions of this article will be made available by the authors, without undue reservation.
